# Magnolol Inhibits High Fructose-Induced Podocyte Inflammation via Downregulation of TKFC/Sp1/HDAC4/Notch1 Activation

**DOI:** 10.3390/ph17111416

**Published:** 2024-10-23

**Authors:** Ziang Zhou, Yumeng Wang, Yu Xing, Shuman Pan, Wanru Wang, Jie Yang, Wenyuan Wu, Jie Zhou, Luyi Huang, Qiongdan Liang, Dongmei Zhang, Lingdong Kong

**Affiliations:** State Key Laboratory of Pharmaceutical Biotechnology, Institute of Chinese Medicine, Nanjing Drum Tower Hospital, School of Life Sciences, Nanjing University, Nanjing 210023, China; zhouziang@smail.nju.edu.cn (Z.Z.); wangyumeng@smail.nju.edu.cn (Y.W.); xingyu@smail.nju.edu.cn (Y.X.); panshuman@smail.nju.edu.cn (S.P.); wangwanru@smail.nju.edu.cn (W.W.); yangjie@smail.nju.edu.cn (J.Y.); wuwenyuan@smail.nju.edu.cn (W.W.); zhoujie@smail.nju.edu.cn (J.Z.); huangluyi@smail.nju.edu.cn (L.H.); liangqiongdan@smail.nju.edu.cn (Q.L.)

**Keywords:** magnolol, podocyte inflammation, TKFC, TNF-α, NICD1, high fructose

## Abstract

Background/Objectives: High fructose has been implicated as an important trigger of kidney inflammation in patients and experimental models. Magnolol, isolated from *Magnolia officinalis*, has an anti-inflammatory effect, but its protective role in podocytes remains underexplored. This study explored the protective effects and underlying mechanism of magnolol against high fructose-induced podocyte inflammation. Methods: The effects of magnolol on high fructose-induced podocyte inflammation were assessed in male Sprague Dawley rats administered 10% (*w*/*v*) fructose water for 12 weeks and heat-sensitive human podocyte cell lines (HPCs) exposed to 5 mM fructose. Podocyte foot processes were examined using transmission electron microscopy. The expression levels of nephrin, podocin, tumor necrosis factor-α (TNF-α), Notch1 intracellular domain (NICD1), triokinase/FMN cyclase (TKFC), specificity protein 1 (Sp1) and histone deacetylase 4 (HDAC4) were determined by Western blot, immunofluorescence and real-time quantitative polymerase chain reaction (qRT-PCR). The chromatin immunoprecipitation (ChIP) assay was performed to evaluate the interaction between Sp1 and the promoter region of HDAC4. Results: Magnolol mitigated the impairment of glomerular filtration function in high fructose-fed rats. Besides, it significantly alleviated the inflammatory responses in glomeruli and HPCs, evidenced by decreased protein levels of TNF-α and NICD1. Increased protein levels of TKFC, Sp1 and HDAC4 were observed in high fructose-stimulated HPCs and rat glomeruli. TMP195, an HDAC4 inhibitor, reduced TNF-α and NICD1 protein levels in high fructose-exposed HPCs. The increased Sp1 was shown to associate with the promoter region of HDAC4, promoting HDAC4 protein expression in high fructose-exposed HPCs. The knockdown of TKFC in HPCs by *TKFC* siRNA decreased Sp1, HDAC4 and NICD1 protein levels, alleviating podocyte inflammatory response. Furthermore, magnolol inhibited TKFC/Sp1/HDAC4/Notch1 activation in vivo and in vitro. Conclusions: Magnolol attenuated high fructose-induced podocyte inflammation possibly through the suppression of TKFC/Sp1/HDAC4/Notch1 activation, providing new evidence for its potential role in podocyte protection.

## 1. Introduction

Clinical studies have reported that drinking at least two beverages containing sugar daily causes high level of urinary albumin excretion [1] and is related to an increased incidence of kidney diseases [2]. It is well known that an excess amount of fructose could cause hyperuricemia [3]. Increased serum uric acid is reported to activate NOD-like receptor pyrin domain containing three (NLRP3) inflammasome, causing renal inflammation in mice after drinking 30% fructose solution for 6 months [4]. In rats fed with high fructose, increased macrophage infiltration and tumor necrosis factor (TNF-α) level in glomeruli are observed [5], further indicating that a high-fructose diet is possibly an inducing factor of glomerular inflammatory response. Podocytes are the important components maintaining glomerular filtration function, entering growth arrest when differentiating into mature cells in adult rodents [6,7]. Podocyte injury is an irreversible step in the development of kidney disease [8]. Thus, investigating podocyte inflammation may possibly elucidate the detailed mechanism of glomerular dysfunction induced by high fructose consumption.

Specificity protein 1 (Sp1) as a significant transcription factor that regulates diverse biological processes [9]. Elevated Sp1 expression is detected in airway inflammation of mouse asthma induced by ovalbumin [10], possibly promoting renal inflammation in a mouse hypertension model induced by angiotensin [11]. These observations indicate that high Sp1 expression activates the downstream inflammatory signaling pathway. Histone deacetylases (HDACs) can mediate downstream gene transcription and control diverse cellular processes [12]. Of note, the expression of HDAC4 is elevated in human podocytes treated with high glucose and advanced glycation end products [13]. HDAC4 gene silencing by lentivirus in an intrarenal delivery way ameliorates renal injury in diabetic rats induced by streptozotocin [13]. Meanwhile, HDAC4 interference mitigates decreased nephrin expression and cell apoptosis in both primary mouse podocytes and immortalized mouse podocytes treated with high glucose [14]. Sp1 can combine with the promoter region of HDAC4 in 293T cells [15]. Increased Sp1 augments DNA methyltransferase 1 expression by binding to its promoter region, possibly contributing to high glucose-induced podocyte injury [16]. Concurrently, HDAC4 mRNA and protein levels are both elevated in SP1-overexpressing MODE-K cells [15]. However, it remains unclear whether Sp1 regulates HDAC4 expression to participate in podocyte inflammatory injury.

Notch1, a membrane receptor of Notch signaling identified in mammals, is activated during mammalian nephrogenesis, and it is silent in mature glomeruli [17]. When activated, Notch1 undergoes three cleavages, and its intracellular domain (NICD1) is transported into the nucleus, which mediates downstream gene transcription [18]. Activated Notch1 is observed in podocytes detected in biopsy samples from diabetic patients complicated with kidney disease, focal segmental glomerulosclerosis and other acquired kidney disorders [19], showing that NICD1 expression in podocytes/glomeruli is positively correlated with albuminuria and glomerulosclerosis in these patients [19]. The overproduction of NICD1 participates in TGF-β1-induced podocyte apoptosis [20]. An overactivated Notch1 pathway may activate NLRP3 inflammasome in the podocytes cultured with puromycin aminonucleoside [21]. In addition, the crosstalk between Notch1 and Toll-like receptor 4 (TLR4) signaling regulates the inflammatory response in IgA nephropathy [22], indicating the potential role of Notch1 pathway activation in podocyte inflammation. Triokinase/FMN cyclase (TKFC), one of the components catalyzing glyceraldehyde phosphorylation in fructose metabolism [23], may function as a switch linking fructolysis with lipogenesis under a fructose diet [24]. TKFC mRNA and protein levels are both elevated in the small intestine of mice orally given 30% fructose solution [25]. However, the interaction between TKFC and Notch1 signaling in podocyte inflammation induced by high fructose has not been illustrated.

Magnolol, a polyphenolic compound, constitutes the main active components of the *Magnolia officinalis* cortex [26]. Magnolol ameliorates upregulated expression levels of interleukin-1β (IL-1β) and TNF-α in lipopolysaccharide-treated U937 cells and in colitis mice induced by dextran sulfate sodium salt, exhibiting anti-inflammatory activity [27]. Magnolol also alleviates proteinuria and urinary creatinine clearance and prevents glomerular enlargement in type 2 diabetes Goto–Kakizaki rats without obesity [28]. However, whether magnolol inhibits high fructose-induced podocyte inflammatory response and the potential molecular mechanism behind this is still unclear. In this research, we intended to investigate the association between high fructose-induced podocyte inflammation and TKFC/Sp1/HDAC4/Notch1 activation and whether magnolol could mitigate the TKFC/Sp1/HDAC4/Notch1 activation in its protection of glomerular podocyte inflammation.

## 2. Results

### 2.1. Magnolol Attenuated Glomerular Inflammatory Injury in High Fructose-Fed Rats with Decreased TNF-α and NICD1

**Figure 1:**
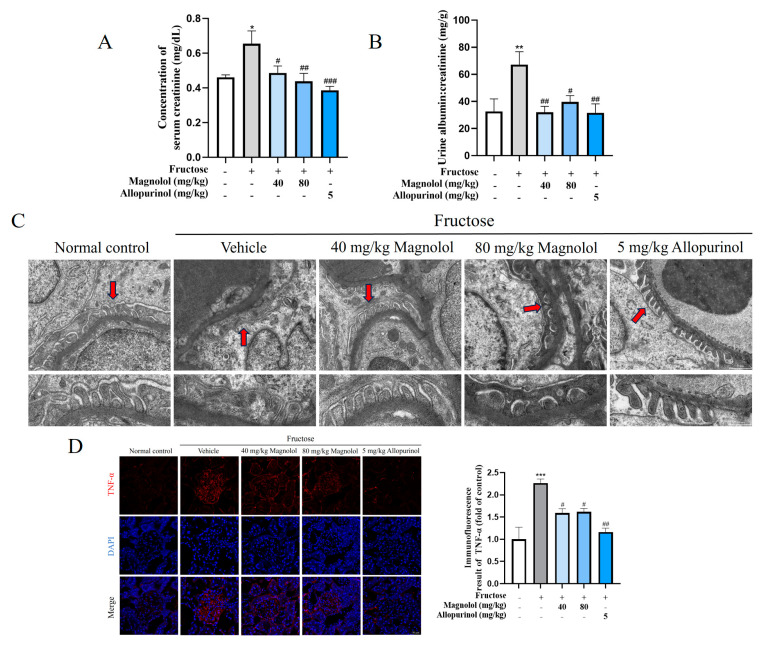

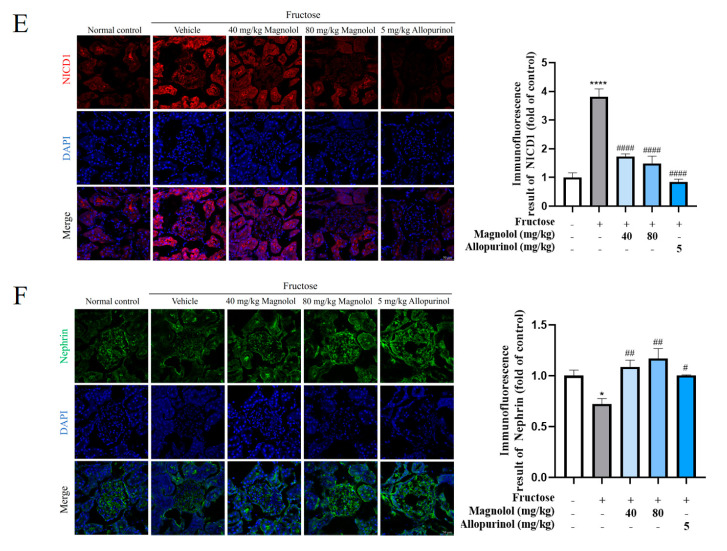
Magnolol attenuated glomerular inflammatory injury with decreased TNF-α and NICD1 rats fed with fructose. (**A**) Serum creatinine levels of rats from five groups (n = 7–8). (**B**) Urine albumin/creatinine ratio of rats from five groups (n = 7–8). (**C**) Transmission electron microscopy observation of podocytes foot process effacement in rats from five groups (n = 3). Podocyte foot processes are pointed out by red arrows (scale bar, upper row: 1 μm; under row: 500 nm). (**D**–**F**) Immunofluorescence images showing TNF-α, NICD1 and nephrin protein levels in the of rat glomeruli from five groups (scale bar, 50 μm, n = 3). All data are shown as mean ± SEM. * *p* < 0.05, ** *p* < 0.01, *** *p* < 0.001, **** *p* < 0.0001 vs. control; ^#^ *p* < 0.05, ^##^ *p* < 0.01, ^###^ *p* < 0.001, ^####^ *p* < 0.0001 vs. fructose.

Magnolol significantly prevented against high fructose-impaired glomerular filtration function, as manifested by the decreased serum creatinine (40 mg/kg: *p* < 0.05, 80 mg/kg: *p* < 0.01) (Figure 1A) and urine albumin/creatinine ratio (UACR) (40 mg/kg: *p* < 0.01, 80 mg/kg: *p* < 0.05) (Figure 1B). Also, allopurinol significantly decreased serum creatinine (*p* < 0.001) and UACR (*p* < 0.01). Podocyte foot processes constitute the slit diaphragms which maintain filtration function. Injured podocytes undergo foot processes effacement, ultimately resulting in proteinuria [29]. Here, magnolol and allopurinol significantly mitigated high fructose-induced foot process effacement (Figure 1C).

Furthermore, magnolol significantly mitigated the expression of inflammatory indicator TNF-α (*p* < 0.05) and Notch1 pathway key factor NICD1 (*p* < 0.0001) in the glomeruli of rats fed with fructose (Figure 1D,E), indicating the attenuation of podocyte inflammation. Nephrin constitutes the glomerular slit diaphragm, and its decreased expression has been implicated as podocyte injury [30,31,32]. Consistently, magnolol significantly reversed the decreased expression of nephrin (*p* < 0.01) in glomeruli of rats fed with high fructose (Figure 1F), showing the prevention of podocyte injury. Similarly, allopurinol reversed the upregulation of TNF-α (*p* < 0.01) and NICD1 (*p* < 0.0001) and the downregulation of nephrin (*p* < 0.05) at protein levels in vivo.

### 2.2. Magnolol Mitigated Inflammatory Injury in Podocytes with Downregulation of TNF-α and NICD1

**Figure 2:**
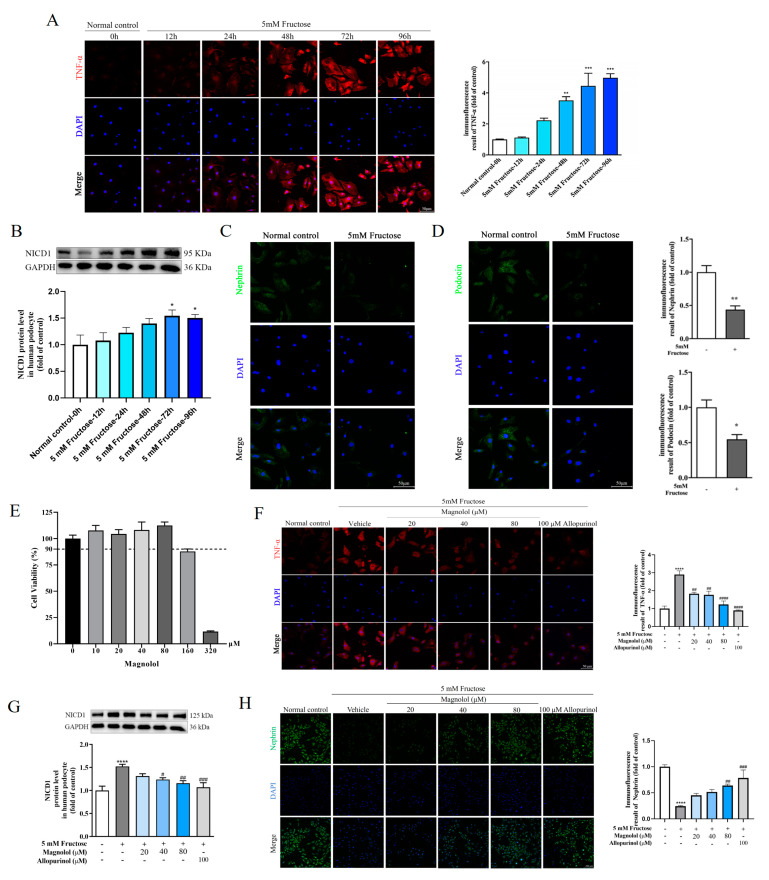
Magnolol mitigated inflammatory injury in podocytes with downregulation of TNF-α and NICD1. (**A**) Immunofluorescence images demonstrating TNF-α expression in HPCs incubated with 5 mM fructose at different time points from 0 to 96 h (scale bar, 50 μm). (**B**) Relative NICD1 protein levels in HPCs treated with 5 mM fructose at different time points from 0 to 96 h, which were normalized to GAPDH. (**C**,**D**) Immunofluorescence images revealing nephrin and podocin expression in HPCs exposed to 5 mM fructose for 72 h (scale bar, 50 μm). (**E**) Cell viability of HPCs exposed to 10, 20, 40, 80, 160, and 320 μM magnolol for 72 h detected by CCK-8 kit. (**F**) Immunofluorescence images showing TNF-α expression in HPCs under different culture conditions (scale bar, 50 μm). (**G**) Relative NICD1 protein levels in HPCs cultured with different conditions, which were normalized to GAPDH. (**H**) Immunofluorescence images manifesting Nephrin protein levels in HPCs under different culture conditions (scale bar, 200 μm). Biologically independent experiments were conducted in triplicate for immunofluorescence assays and six times for Western blot assays. All data are shown as mean ± SEM. * *p* < 0.05, ** *p* < 0.05, *** *p* < 0.001, **** *p* < 0.0001 vs. control; ^#^ *p* < 0.05, ^##^ *p* < 0.01, ^###^ *p* < 0.001, ^####^ *p* < 0.0001 vs. fructose.

In differentiated HPCs treated with 5 mM fructose for 0, 12, 24, 48, 72, and 96 h, the protein levels of TNF-α was increased time-dependently by immunofluorescence analysis (Figure 2A). Simultaneously, NICD1 protein levels were obviously increased after 48, 72 and 96 h stimulation by fructose (Figure 2B). In subsequent experiments, we chose 72 h as the high fructose-exposed time for constructing podocyte models with a significant downregulation of nephrin and podocin (Figure 2C,D).

We analyzed the toxicity of magnolol on differentiated HPCs by cell counting kit-8 (CCK-8) assay at 10, 20, 40, 80, 160, and 320 μM for 72 h. In further experiments, 20, 40 and 80 μM of magnolol were chosen as the work doses, as the cell viability under these concentrations was above 90% (Figure 2E). To verify whether magnolol could attenuate podocyte inflammatory response, 5 mM fructose-stimulated differentiated HPCs were treated with 20, 40, and 80 μM magnolol and 100 μM allopurinol for 72 h. Immunofluorescence analysis manifested that magnolol ameliorated high fructose-induced overexpression of TNF-α (20 and 40 μM: *p* < 0.01, 80 μM: *p* < 0.0001) (Figure 2F). Western blot assay demonstrated that magnolol downregulated NICD1 protein levels (40 μM: *p* < 0.05, 80 μM: *p* < 0.01) (Figure 2G). Meanwhile, magnolol ameliorated the downregulation of nephrin (80 μM: *p* < 0.01) induced by fructose (Figure 2H). Similarly, 100 μM allopurinol reversed the upregulation of TNF-α (*p* < 0.0001) and NICD1 (*p* < 0.001) and the downregulation of nephrin (*p* < 0.001) induced by high fructose. These data further confirmed that magnolol attenuated podocyte inflammatory injury induced by high fructose.

### 2.3. High Sp1 Expression Promoted HDAC4 Expression and Then Drove Notch1 Signaling Pathway in High Fructose-Exposed Podocytes

**Figure 3:**
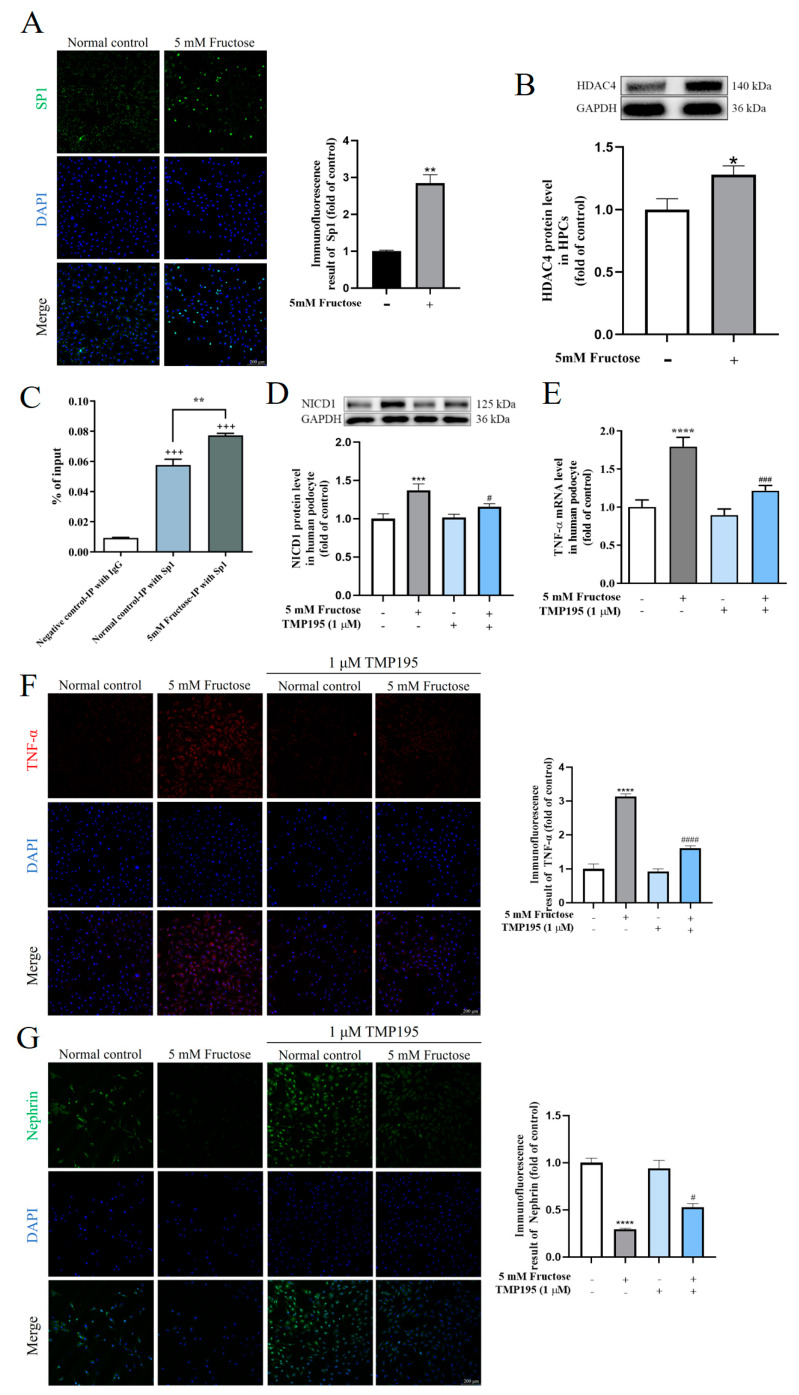
Increased Sp1 promoted HDAC4 expression and then drove the Notch1 signaling pathway in podocytes exposed to high fructose for 72 h. (**A**) Immunofluorescence images showing Sp1 expression in HPCs (scale bar, 200 μm). (**B**) Relative HDAC4 protein levels in HPCs, which were normalized to GAPDH. (**C**) Chromatin IP analysis of Sp1 and HDAC4 promoter region binding in HPCs. (**D**) Relative NICD1 protein levels in HPCs treated with or without 5 mM fructose in the presence or absence of TMP195 (1 μM) for 72 h, respectively, which were normalized to GAPDH. (**E**) Relative mRNA levels of TNF-α in HPCs cultured with different conditions, which were normalized to β-actin. (**F**,**G**) Immunofluorescence images demonstrating TNF-α and Nephrin expression in HPCs under different culture conditions (scale bar, 200 μm). Biologically independent experiments were conducted in triplicate for immunofluorescence and ChIP assays and six times for Western blot assays. All data are shown as mean ± SEM. * *p* < 0.05, ** *p* < 0.01, *** *p* < 0.001, **** *p* < 0.0001 vs. control; ^+++^ *p* < 0.001 vs. negative control; ^#^ *p* < 0.05, ^###^ *p* < 0.001, ^####^ *p* < 0.0001 vs. fructose.

In HPCs cultured with 5 mM fructose, high Sp1 expression was observed with more Sp1 translocating into the cell nucleus (Figure 3A). Meanwhile, Western blot assay showed that HDAC4 protein levels were also increased (Figure 3B).

Next, it was verified by ChIP assay whether Sp1 could combine with sites in the promoter region of HDAC4. As shown in Figure 3C, many DNA sequences in the promoter region of HDAC4 were detected containing the binding sites of Sp1 in the chromatin samples precipitated by anti-Sp1 antibody. After 5 mM fructose stimulation, the combination of Sp1 with the sites in the HDAC4 promoter was apparently augmented, which promoted the expression of HDAC4 in differentiated HPCs.

To further analyze the effect of HDAC4 on inflammatory injury in podocytes, differentiated HPCs were treated with TMP195 (1 μM), which is an HDAC4 inhibitor. After 72 h incubation with TMP195, the inhibition of HDAC4 activity by TMP195 significantly reduced the NICD1 protein levels in fructose-stimulated podocytes (Figure 3D), indicating the possible upstream regulatory effect of HDAC4 on NICD1 to activate the Notch1 signaling pathway. Meanwhile, TMP195 significantly decreased the expression of TNF-α in differentiated HPCs cultured with 5 mM fructose for 72 h (Figure 3E,F), while the protein levels of nephrin were increased (Figure 3G), indicating that the inhibition of HDAC4 activity restored high fructose-induced inflammatory injury in differentiated HPCs.

### 2.4. Knockdown of TKFC Decreased HDAC4 Expression and Notch1 Pathway Activation to Alleviate Podocyte Inflammatory Response

**Figure 4:**
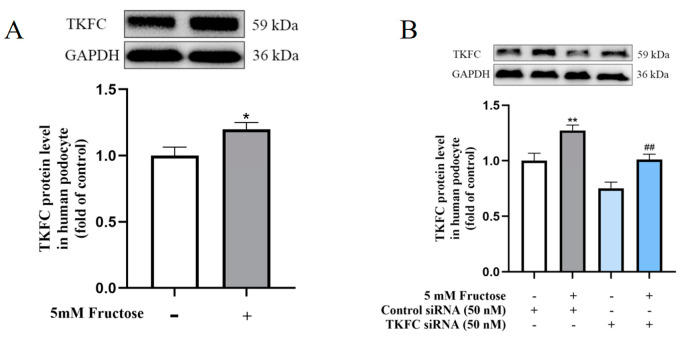

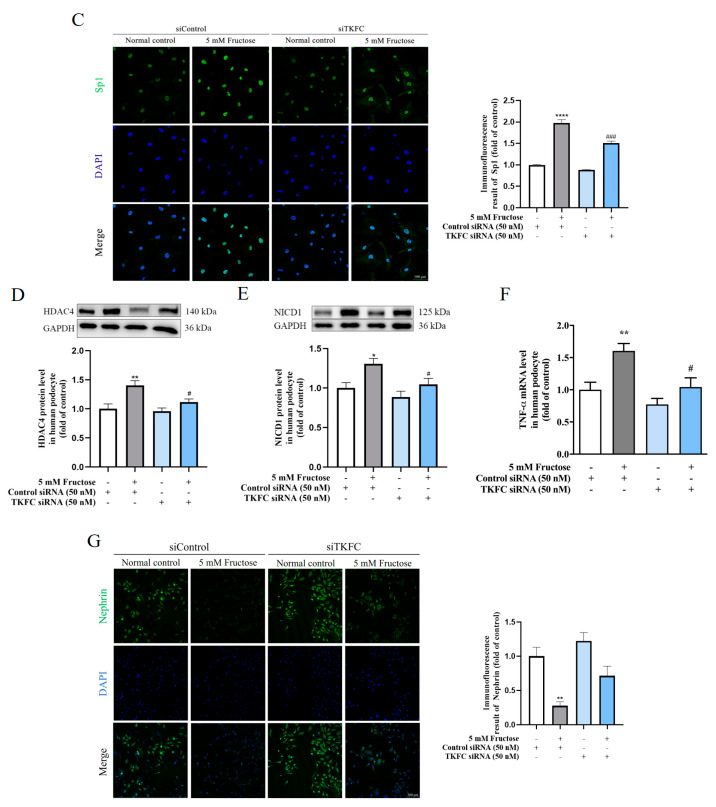
Knockdown of TKFC decreased HDAC4 expression and Notch1 pathway activation to alleviate podocyte inflammatory response. (**A**) Relative TKFC protein levels in HPCs. The results are shown as mean ± SEM. * *p* < 0.05 vs. control. (**B**) Relative TKFC protein levels in differentiated HPCs transfected with *TKFC* or control siRNA and cultured with or without 5 mM fructose for 72 h, respectively. (**C**) Immunofluorescence images demonstrating Sp1 expression in HPCs treated under different conditions (scale bar, 100 μm). (**D**,**E**) Relative protein levels of HDAC4 and NICD1 in HPCs treated under different conditions. (**F**) Relative mRNA levels of TNF-α in HPCs treated under different conditions. (**G**) Immunofluorescence images showing nephrin levels in HPCs treated under different conditions (scale bar, 200 μm). Relative protein levels of TKFC, HDAC4 and NICD1 were normalized to GAPDH and β-actin was utilized to normalize the mRNA expression of TNF-α. Biologically independent experiments were conducted in triplicate for immunofluorescence assays and six times for Western blot assays. All data are shown as mean ± SEM. * *p* < 0.05, ** *p* < 0.01, **** *p* < 0.0001 vs. control + control siRNA (except Figure 4A); ^#^ *p* < 0.05, ^##^ *p* < 0.01, ^###^ *p* < 0.001 vs. fructose + control siRNA.

The TKFC protein was augmented in HPCs exposed to high fructose for 72 h (Figure 4A). To explore the regulatory mechanism of TKFC in fructose-induced inflammatory response, *TKFC* siRNA was transfected into differentiated HPCs. *TKFC* siRNA significantly reversed the increase in TKFC in HPCs induced by high fructose (Figure 4B). As a transcription factor, Sp1 functions in the nucleus. After the knockdown of TKFC, the nuclear proportion of Sp1 was decreased in differentiated HPCs cultured with 5 mM fructose (Figure 4C). As expected, the protein levels of HDAC4 were declined in differentiated HPCs with fructose exposure in the knockdown of TKFC (Figure 4D). Furthermore, *TKFC* siRNA decreased NICD1 protein levels (Figure 4E) and TNF-α mRNA levels (Figure 4F) in differentiated HPCs cultured with 5 mM fructose, while the decrease in nephrin protein levels was improved by TKFC knockdown (Figure 4G). These results suggested that TKFC upregulated the expression of Sp1 and HDAC4, thus promoting Notch1 signaling activation and inflammatory response in differentiated HPCs stimulated with high fructose.

### 2.5. Magnolol Attenuated High Fructose-Induced Inflammatory Injury Possibly by Inhibiting TKFC/Sp1/HDAC4/Notch1 Pathway in Glomeruli of Rats and HPCs

**Figure 5:**
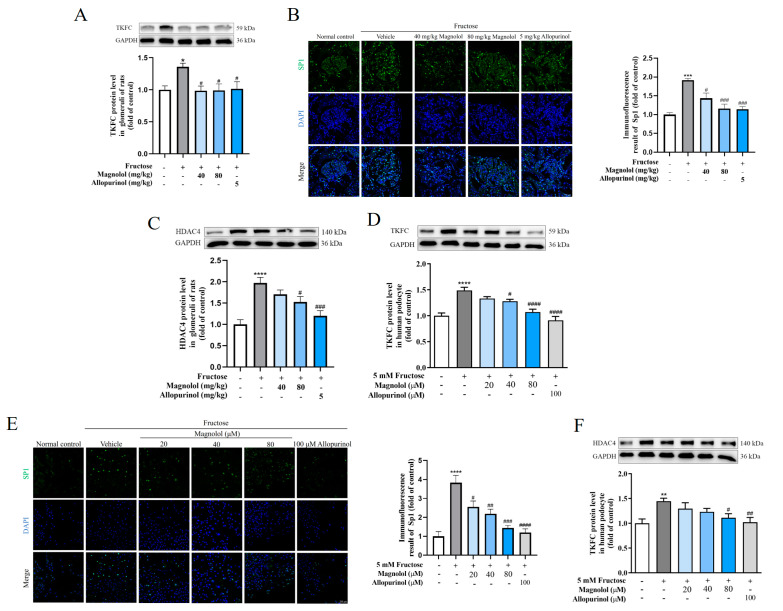
Magnolol alleviated high fructose-induced inflammatory injury by inhibiting the TKFC/Sp1/HDAC4/Notch1 pathway in the glomeruli of rats and HPCs. (**A**) Relative TKFC protein levels in the rat glomeruli from five groups (n = 6). (**B**) Immunofluorescence images showing Sp1 expression in the rat glomeruli from five groups (scale bar, 50 μm). (**C**) Relative HDAC4 protein levels in the rat glomeruli from five groups. (**D**) Relative TKFC protein levels in HPCs under different culture conditions. (**E**) Immunofluorescence images demonstrating Sp1 expression in HPCs under different culture conditions (scale bar, 200 μm). (**F**) Relative HDAC4 protein levels in HPCs under different culture conditions. GAPDH was employed to normalize the protein levels of TKFC and HDAC4. Biologically independent experiments were conducted in triplicate for immunofluorescence assays and six times for Western blot assays. All data are shown as mean ± SEM. * *p* < 0.05, ** *p* < 0.01, *** *p* < 0.001, **** *p* < 0.0001 vs. control; ^#^ *p* < 0.05, ^##^ *p* < 0.01, ^###^ *p* < 0.001, ^####^ *p* < 0.001 vs. fructose.

To verify whether the TKFC/Sp1/HDAC4/Notch1 pathway was the potential mechanism by which magnolol prevented podocyte inflammatory injury, Western blot and immunofluorescence analysis were employed to detect the protein levels of TKFC, Sp1 and HDAC4 in rat glomeruli and HPCs. Magnolol decreased the protein levels of TKFC (*p* < 0.05), Sp1 (40 mg/kg: *p* < 0.05, 80 mg/kg: *p* < 0.001) and HDAC4 (80 mg/kg: *p* < 0.05) in the glomeruli of rats with a high-fructose diet (Figure 5A–C). Similarly, allopurinol decreased the protein levels of TKFC (*p* < 0.05), Sp1 (*p* < 0.001) and HDAC4 (*p* < 0.001) in the glomeruli of rats with a high-fructose diet. Meanwhile, magnolol obviously downregulated the protein levels of TKFC (40 μM: *p* < 0.05, 80 μM: *p* < 0.0001), Sp1 (20 μM: *p* < 0.05, 40 μM: *p* < 0.01, 80 μM: *p* < 0.001) and HDAC4 (80 μM: *p* < 0.05) (Figure 5D–F) in podocytes treated with high fructose with a dose-dependent effect. Allopurinol also reduced the protein levels of TKFC (*p* < 0.0001), Sp1 (*p* < 0.0001) and HDAC4 (*p* < 0.01) in this cell model.

Hence, magnolol significantly attenuated high fructose-induced increased protein levels of TKFC, Sp1, and HDAC4 in rat glomeruli and HPCs. Since magnolol suppressed NICD1 expressions in rat glomeruli and HPCs (Figure 1E and Figure 2G), it was possible that magnolol mitigated high fructose-induced inflammatory injury by inhibiting the TKFC/Sp1/HDAC4/Notch1 pathway. In this study, allopurinol had similar effects (Figure 1, Figure 2 and Figure 5).

## 3. Discussion

Fructose consumption has conspicuously soared in the last several decades, which is considered as a trigger for glomerular dysfunction [33]. Our previous studies demonstrated that high fructose contributed to podocyte injury both in vivo and in vitro [34,35]. In this research, TNF-α, an inflammatory factor, was significantly increased in high fructose-stimulated differentiated HPCs and rat glomeruli. TKFC catalyzes glyceraldehyde phosphorylation in fructose metabolism, completing the final step in fructolysis [36]. We found that high fructose upregulated TKFC protein levels in differentiated HPCs and rat glomeruli, which were inhibited by magnolol in its attenuation of podocyte injury.

Elevated Sp1 expression is reported in the kidney of patients with glomerulonephritis [37,38] and podocytes in db/db mice [16]. In this research, Sp1 expression levels were augmented in HPCs and rat glomeruli treated with high fructose, whose translocation to the nuclei was detected by immunofluorescence staining experiments. ChIP assay verified that Sp1 could combine with the promoter region of HDAC4 with the transcription of HDAC4 promoted in differentiated HPCs. The expression level of HDAC4 is upregulated in the kidneys of diabetic rats induced by streptozotocin, db/db mice and renal samples from diabetic nephropathy patients [13]. Also, HDAC4 is significantly elevated in lipopolysaccharides-exposed podocytes [39]. SP1 is enriched within the promotor region of HDAC4, and dual-luciferase reporter assay demonstrates that the luciferase activity of the HDAC4 promoter is augmented upon SP1 overexpression in 293T cells transfected with pcDNA3.1-SP1 expression vector [15]. Here, in fructose-stimulated HPCs, high HDAC4 expression and the transcriptional regulation effect of Sp1 on HDAC4 were observed, indicating that the nucleic translocation of Sp1 might increase HDAC4 expression induced by high fructose. Therefore, TMP195, an HDAC4 inhibitor, was used to study the role of Sp1/HDAC4 activation in podocyte inflammation induced by high fructose. TMP195 significantly inhibited TNF-α production and increased nephrin levels in this cell model, suggesting that Sp1/HDAC4 suppression might alleviate podocyte inflammation induced by high fructose exposure.

Although Notch1 is seldom expressed in the kidneys of healthy adults, the re-expression and activation of Notch1 are found in podocytes and tubular cells of patients diagnosed with kidney diseases, indicating that its activation may be essential for the progression of diabetic nephropathy [40]. The Notch1 signaling pathway also plays a part in the occurrence of renal inflammatory response. It is overactivated in podocytes of lupus nephritis patients [21]. Notch1 activation is detected in Gremlin-injected mice with renal inflammatory cell infiltration and an overexpression of proinflammatory cytokines [41]. Anti-inflammatory effects of HDACs inhibitors in mouse and human kidney have been reported [19,42,43]. This study demonstrated that the protein levels of NICD1, the Notch1 active form, were apparently increased in HPCs and rat glomeruli with high fructose stimulation, accompanied by an elevated expression of TNF-α, indicating that the Notch1 signaling pathway was activated to induce inflammatory response by high fructose. Moreover, the upregulation of NICD1 was significantly reduced by TMP195, suggesting the regulatory effect of HDAC4 on Notch1 signaling. Further experiments revealed that TKFC regulated the activation of Sp1/HDAC4 and Notch1 signaling. In high fructose-stimulated HPCs, TKFC knockdown decreased the nuclear proportion of Sp1, protein levels of HDAC4 and NICD1. Consistently, high fructose-induced TNF-α overexpression and nephrin downregulation were restored by *TKFC* siRNA transfection, manifesting the amelioration of inflammatory injury and TKFC/SP1/HDAC4/Notch1 activation in high fructose-induced podocyte inflammation. We further conducted a Co-IP assay to determine whether TKFC could directly interact with Sp1. However, the result showed that there was no direct interaction between TKFC and Sp1. The mechanism by which TKFC regulated the expression of Sp1 needs to be further explored.

Since the inflammatory response can be triggered by multiple mechanisms, it is possible that the proposed TKFC/SP1/HDAC4/Notch1 pathway may potentially interact with other inflammatory pathways to mediate high fructose-induced podocyte inflammation. The nuclear translocation of Sp1 in cooperation with nuclear factor-kappaB (NF-κB) augments monocyte chemoattractant protein 1 production in carboxymethyllysine-stimulated mouse differentiated podocytes, which facilitates macrophages recruitment [44]. Both Sp1 and NF-κB binding sites exist in the IL-6 promotor region demonstrated in IB3-1 cells infected with *Pseudomonas aeruginosa* [45], indicating that Sp1 may cooperate with NF-κB to mediate inflammation. Sp1 can also combine with the promoter region of NLRP3 in mouse vascular endothelial cell line C166 treated with oxidized low-density lipoprotein [46]. The activation of NLRP3 results in apoptosis and cytoskeleton changes in mouse podocytes treated with 30 mM glucose [47], so it is possible that Sp1 may promote the expression of NLRP3 to induce podocyte inflammation in our high-fructose model. HDAC4 may contribute to NF-κB activation dependent on ROS production, causing vascular inflammation in rat mesenteric arterial smooth muscle cells exposed to TNF-α [48]. Also, HDAC4 deacetylates signal transducer and activator of transcription 1, permitting its phosphorylation and activation, possibly contributing to inflammation and apoptosis in glucose-exposed human podocytes [13]. Notch1 signaling activates the NLRP3 inflammasome in puromycin aminonucleoside-stimulated mouse podocytes [21]. The blockage of Notch1 signaling by *N*-[*N*-(3,5-Difluorophenacetyl)-l-alanyl]-*S*-phenylglycine *t*-butyl ester suppresses the expressions of phosphorylated NF-κB p65 and inflammatory cytokines in lipopolysaccharide-stimulated human podocytes [22]. Therefore, the TKFC/SP1/HDAC4/Notch1 signaling reported in this study may possibly cooperate with other inflammatory pathways to mediate high fructose-induced podocyte inflammation, which needs to be further explored.

**Figure 6:**
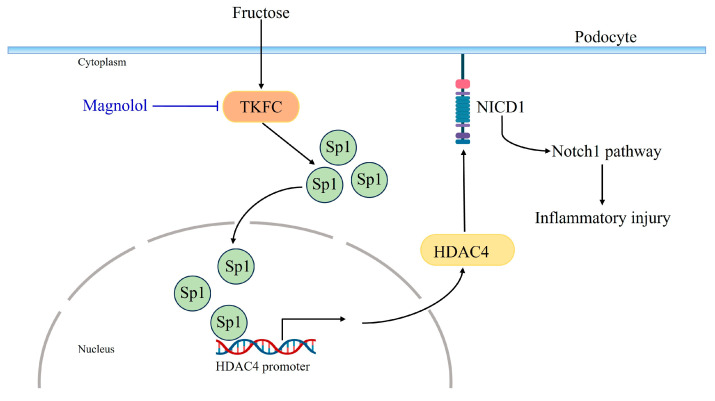
The proposed mechanism by which magnolol mitigated podocyte inflammation induced by high fructose.

The ethanol extract of *M. officinalis* improves renal function with mitigated inflammation and oxidative stress in mice fed with a high-fat diet [49]. Magnolol, as one of the main active ingredients of *M. officinalis*, attenuates glomerular filtration dysfunction in Goko–Kakizaki rats treated with 100 mg/kg magnolol [28]. Also, magnolol significantly reduces serum TNF-α levels as well as protein levels of renal TNF-α in ischemia–reperfusion rats [50]. It improves nephritis symptoms in a systemic lupus erythematosus model of mice [51]. However, few studies revealed the role of magnolol in attenuating podocyte injury. We firstly reported that magnolol alleviated glomerular podocyte injury in rats fed with high fructose, as manifested by the decreased serum creatinine and UCAR, and the mitigation of foot process effacement in the glomeruli of high fructose-fed rats. More importantly, magnolol was found to reduce podocyte TKFC protein levels in HPCs and rat glomeruli with high fructose exposure, indicating that intervention in the dysregulation of fructose-metabolizing enzymes may help ameliorate inflammatory injury in podocytes. Further experiments revealed that magnolol effectively suppressed the activation of Notch1 signaling, protein levels of TNF-α and NICD1, as well as upregulated nephrin protein levels in HPCs and rat glomeruli stimulated by high fructose. Moreover, it downregulated Sp1 and HDAC4 protein levels in this cell and animal models. Considering the potential mechanism of TKFC/SP1/HDAC4/Notch1 in podocyte inflammation under high fructose exposure, we suggested that magnolol might prevent high fructose-induced podocyte inflammation through suppressing TKFC/Sp1/HDAC4/Notch1 activation (Figure 6). Regarding the dose-dependent effects of magnolol, in the cell experiments, it could be seen that 80 μM magnolol mitigated high fructose-induced podocyte inflammation more significantly than 20 and 40 μM magnolol. Therefore, in this research, the nephroprotective effects of magnolol against podocyte injury induced by high fructose in vitro were possibly dose-dependent.

Magnolol mitigates STAT3 pathway activation and inhibits downstream inflammation-associated gene expression in bovine aortic endothelial cells stimulated with IL-6 [52]. This compound inhibits the expression of TLR4, which suppresses mitogen-activated protein kinase and the NF-κB pathway in lipopolysaccharide-stimulated RAW264.7 cells with a decreased production of several inflammatory factors, including TNF-α [53]. In addition, magnolol suppresses NLRP3 inflammasome activation, which mitigates the expression of IL-1β in the liver of BALB/c mice gavaged with absolute ethanol [54]. It increases nuclear factor erythroid 2-related factor 2, mitigating the expression of IL-6 and IL-8 in human gingival fibroblasts treated with advanced glycation end-products [55]. The TKFC/SP1/HDAC4/Notch1 signaling may interact with other pathways to mediate podocyte inflammation; further study is needed to explore the major pathway mediated by magnolol in its mitigation of podocyte inflammation induced by high fructose.

However, this study has some inadequacies that need to be improved in further research. The mechanism by which TKFC regulated the increased expression of Sp1 has not been completely expounded. Also, to support the effect of the TKFC/Sp1/HDAC4/Notch1 signaling on high fructose-induced podocyte inflammation, overexpressing TKFC or specific components of the pathway will solidify the proposed mechanism. In addition, it is apparent that the differentiation of HPCs does not resemble the normal development of podocytes in vivo, and primary podocytes are assumed to retain their phenotypes resembling the podocytes in vivo because of short-term culture. Therefore, primary podocytes could be used to solidify the proposed mechanism in further research.

## 4. Materials and Methods

### 4.1. Animal Treatment

Forty male Sprague–Dawley rats aged around 8 weeks old and weighing range from 210 to 230 g were acquired from the Experimental Animal Centre of Zhejiang Province (Hangzhou, China). All rats were acclimated under standard conditions (room temperature: 23 ± 1 °C; humidity: 50 ± 5%; a 12 h light/dark cycle) for a week. After acclimation, 40 rats were separated randomly into two groups: 8 rats in the control group and 32 rats in the fructose group. The fructose group was provided with 10% fructose (Xiwang Sugar Industry, Binzhou, Shandong, China) water, and rats in both groups took standard chow and water freely. After the consumption of fructose water for 6 weeks, the fructose group was further separated into four subgroups with 8 rats in each group: fructose group; 40 mg/kg magnolol group; 80 mg/kg magnolol group and 5 mg/kg allopurinol group. Both magnolol (≥98%, Yuanye Bio-Technology, Shanghai, China) and allopurinol (A8003, Sigma-Aldrich, St. Louis, MO, USA) were given by oral gavage administration. The four subgroups were still given 10% fructose water for the next 6 weeks. Clinical study has shown that allopurinol delays the progression of kidney diseases in patients with chronic kidney disease [56]. Our previous study showed that allopurinol inhibited NLRP3 inflammasome activation and decreased IL-1β in the glomeruli of rats fed with high fructose [57]. Hence, we selected allopurinol as a positive drug.

After the treatment by magnolol or allopurinol for 6 weeks, metabolic cages were adopted to collect 24 h urine samples. Impurities were removed from the obtained urine samples by centrifugation (3000 rpm, 4 °C, 30 min). The supernatant was utilized for further biochemical analysis.

The protocol for animal experiments was checked and approved by the Science and Technology Ethics Committee of Nanjing University (IACUC-2303013) with an animal use permit (SYXK(Su)2019-0056).

### 4.2. Serum and Kidney Sample Collection

Rats were anesthetized by pentobarbital sodium (Tianjin Yifang Technology, Tianjin, China) and intraperitoneally injected with a dose of 40 mg/kg. The rat abdominal aorta was punctured, which is where the blood samples were harvested. Blood was left to stand for 30 min at room temperature and centrifuged at 2500 rpm for 30 min at 4 °C. The supernatant serum was transferred into new EP tubes.

Renal medulla portions were excised from rat kidneys, and cortex portions were dissected into little pieces which were then filtrated with pressure through 180 μm and 150 μm stainless mesh with cooled Hank’s Balanced Salt Solution (C14175500BT, Gibco, Grand Island, NY, USA) for three times, respectively. After centrifugation (2500 rpm, 4 °C, 30 min), most of the residual sediment from the collected solution was considered as glomeruli and stored at −80 °C.

Creatinine (C011-2) levels in serum and urine as well as urine microalbumin (H127A) concentrations were quantified by utilizing corresponding kits (Jiancheng, Nanjing, China), respectively.

### 4.3. Cell Culture and Treatment

The heat-sensitive HPCs whose passage numbers ranged from 10 to 25 were a gift from Dr. Zhi-Hong Liu (Nanjing General Hospital of Nanjing Military Command, Nanjing, China). The temperature-sensitive SV40-T gene has been transfected into HPCs, which allows HPCs to proliferate at 33 °C in complete RPMI-1640 medium (L210KJ, Yuanpei Bio-Technology, Shanghai, China) with recombinant interferon-γ (IFN-γ) (485-MI-100, R&D System, Minneapolis, MN, USA) added to a concentration of 10 U/mL [58]. After being transferred to 37 °C, HPCs cease proliferation and are induced to differentiate without IFN-γ [58].

Differentiated HPCs were cultured with complete RPMI-1640 medium (C11875500CP, Thermo Fisher Scientific, Schwerte, Germany) supplemented with 5 mM fructose or not (F0127, Sigma-Aldrich, St. Louis, MO, USA) in the presence or absence of magnolol (20, 40 and 80 μM) or 100 μM allopurinol for 72 h. The doses of fructose and allopurinol were determined according to our previous studies [59,60]. Magnolol was dissolved in DMSO (Yuanye Bio-Technology, Shanghai, China) as the stock solution. The applicable doses of magnolol were determined by the CCK-8 assay. After cultivation for 72 h, differentiated HPCs were harvested for the subsequent experiments. Before adding fructose in the culture medium to induce HPCs injury, TMP195 (1 μM) (S82815, Yuanye Bio-Technology, Shanghai, China) was supplemented 1 h in advance.

### 4.4. Quantitative Real-Time PCR (qRT-PCR) Assay

The isolation of total RNA from HPCs was conducted by employing Trizol reagent (R401-01-AA, Vazyme, Nanjing, China) in accordance with its manufacture’s instructions. The sequences of the primers (Generay Biotechnology, Shanghai, China) used in this research are shown in Table 1. Reverse transcription action of the total RNA obtained was conducted by utilizing 5× HiScript II Select qRT SuperMix (R222-01, Vazyme). The qRT-PCR analysis for each sample was conducted in triplicate with 2 × iTaqTm Universal SYBR Green Supermix (Q321-01, Vazyme) and data were recorded by CFX Manager Software version 3.0 (Bio-Rad, Hercules, CA, USA). The relative expressions of the genes studied in this research were normalized to the expression level of β-actin and measured by the Ct (2^−ΔΔCt^) method to obtain quantitative results.

### 4.5. Western Blot Analysis

Cell lysis buffer (P0013, Beyotime, Nanjing, China) containing 1 mM phenylmethanesulfonyl fluoride (P105539, Aladdin, Shanghai, China) was added in 6-well plates where HPCs were cultured. After being kept on ice for 30 min, the HPCs were scraped thoroughly from the plates, and the lysate was drawn into 1.5 mL Eppendorf (EP) tubes. Then, these tubes were centrifuged to obtain the supernatant under a fixed condition (12,000× *g*, 4 °C, 15 min), which was consistent throughout this research. The concentrations of protein in each sample were detected by Pierce™ BCA protein assay kit (23225, Thermo Fisher Scientific, Waltham, MA, USA). After the corresponding volume of 5× loading buffer and mercaptoethanol were added, the final samples were mixed adequately and kept in boiling water for 10 min.

Equal quantities of protein derived from different samples was loaded on 7.5% or 10% SDS-PAGE gels. After separation, all of the protein was transferred onto polyvinylidene difluoride membranes (IPVH100010, Millipore, Boston, MA, USA). After blocking, the membranes were incubated with corresponding primary antibodies at respective working concentrations according to their instruction books. All of the primary antibodies utilized in this research are listed below: anti-TKFC (1:2000, 12224-1-AP, Proteintech, Chicago, IL, USA), anti-HDAC4 (1:500, ab12172, Abcam, Cambridge, MA, USA), anti-NICD1 (1:500, ab52301, Abcam), anti-HDAC4 (1:500, A13510, Abclonal, Wuhan, China), anti-cleaved Notch1 (1:1000, A19090, Abclonal) and anti-GAPDH (1:5000, AP0066, Bioworld, Nanjing, China). After overnight incubation at 4 °C, HRP-conjugated anti-rabbit IgG antibody (1:5000, SA00001-2, Proteintech) was employed to incubate membranes for 1 h at indoor temperature. Immunoreactive bands at corresponding positions of membranes were visualized by using enhanced chemiluminescence liquid (Tanon, Shanghai, China), and the quantification of gray value was realized by ImageJ software (version 1.46r).

### 4.6. TKFC Knockdown by Small Interfering RNA (siRNA)

The nucleotide sequences of *TKFC* siRNA and negative control (BIotend, Shanghai, China) utilized in this research are shown in Table 2. The whole experiment was performed in accordance with the instruction book provided by BIotend. When the differentiated HPCs grew to 50% confluence, they were transfected with *TKFC* or control siRNA in Opti-MEM|Reduced-Serum Medium (31985070, Thermo Fisher Scientific, Schwerte, Germany) at the concentration of 50 nM, and the process was conducted using Lipofectamine 2000 (11668-019, Invitrogen, Carlsbad, CA, USA), respectively. After 6 h of transfection, the medium was replaced with complete RPMI-1640 medium in the presence or absence of 5 mM fructose, where HPCs were further cultured for 72 h. The protein levels of TKFC were detected by Western blot assay to measure the transfection efficiency after which the effects of TKFC knockdown on HPCs were explored.

### 4.7. Immunofluorescence Assay

After being cultured under different conditions for 72 h, HPCs were washed three times with cold PBS solution to clear away culture medium. Then, podocytes were fixed by 4% paraformaldehyde solution for 20 min at indoor temperature. After another three washes, HPCs were blocked with QuickBlock™ Blocking Buffer (P0235, Beyotime, Nanjing, China) for 20 min at indoor temperature. HPCs were incubated overnight at 4 °C with corresponding primary antibodies which were diluted with appropriate proportions. The primary antibodies employed in this research included anti-Sp1 (1:200, 21962-1-AP, Proteintech, Chicago, IL, USA), anti-TNF-α (1:200, 60291-1-Ig, Proteintech), anti-nephrin (1:100, ab58968, abcam, Cambridge, MA, USA), anti-podocin (1:100, ab50399, abcam) and anti-NICD1 (1:100, A19090, Abclonal, Wuhan, China). Afterwards, the primary antibody was sucked out and discarded. After 3 washes, the corresponding secondary antibodies were added to accomplish incubation with HPCs for 1 h at indoor temperature, including Alexa Fluor 647 goat anti-mouse (1:500, A21235, Invitrogen, Shanghai, China) and Alexa Fluor 555 goat anti-rabbit (1:500, A21428, Invitrogen). 4, 6-diamidino-2-phenylindole (DAPI) staining solution (P0131, Beyotime, Nanjing, China) was utilized to stain the HPCs nuclei and delay the progress of fluorescence quenching. The procedure of immunofluorescence assay applied to glomeruli was analogous. A confocal laser scanning microscope (Leica TCS SP8-MaiTai M, Wetzlar, Germany) was employed to detect the intensity of fluorescence in every sample.

### 4.8. Chromatin Immunoprecipitation (ChIP) Assay

ChIP assay was conducted by employing the kit (#9003, Cell Signaling Technology, Cambridge, MA, USA), and operating steps were executed according to the manufacturer’s instructions. Then, 37% formaldehyde was added to reach the concentration of 1% in the culture medium to operate a cross-link reaction at 37 °C for 10 min. Next, HPCs were incubated with glycine at the concentration of 0.125 mol/L for 5 min at room temperature to cease the cross-link reaction. After being washed two times with ice-cold PBS, HPCs were collected into centrifuge tubes containing PBS and a protease inhibitor cocktail and then centrifuged at 2000× *g* for 5 min at 4 °C, which was followed by nuclear preparation and chromatin digestion. Briefly, the nuclei were pelleted by centrifugation and then digested with micrococcal nuclease for 20 min, after which the DNA was split into segments around 150–900 bp in length. Then, EDTA was added to cease the digestion process. After centrifugation at 16,000× *g* for 1 min at 4 °C, the nuclei were sonicated to disrupt the nuclear membrane, which was followed by centrifugation for 10 min to clarify the lysate. The supernatant and the cross-linked chromatin preparation can be stored at −80 °C. Subsequently, chromatin digestion to obtain purified DNA ranging in length from 150 to 900 bp and concentration analysis were conducted. Anti-Sp1 antibody and IgG antibody (Normal Rabbit IgG #2729) were added into chromatin samples, which were incubated overnight at 4 °C with rotation. Then, each sample was added with Protein G Magnetic Beads and rotated at 4 °C for 2 h to facilitate full incubation. Chromatin washing and elution were performed for cross-linked chromatin preparation. Briefly, the DNA was purified by repeated centrifugation and washed using a spin column. Finally, the quantity of DNA was measured by real-time qPCR by employing SYBR Green I dye.

### 4.9. CCK-8 Assay

A standard CCK-8 assay was conducted to detect the viability of HPCs. HPCs were seeded into a 96-well plate and induced to differentiate. After differentiation, HPCs were cultured in fresh RPMI-1640 medium with diverse concentrations of magnolol (0, 10, 20, 40, 80, 160, and 320 μM) at 37 °C for 72 h. The effect of magnolol on the viability of differentiated HPCs was detected with the CCK-8 kit (C0039, Beyotime, Nanjing, China) according to its instructions.

### 4.10. Statistical Analysis

Data within this research were all shown as the mean ± SEM. An unpaired *t* test was employed to compare the data between two groups. One-way ANOVA with Dunnett’s post hoc test was utilized to conduct statistical comparisons among more than two groups through the use of GraphPad Prism 8 (San Diego, CA, USA). The statistical difference was perceived to be significant when the *p* value was lower than 0.05.

## 5. Conclusions

In conclusion, the present study firstly reported a new mechanism that TKFC upregulation increased Sp1 expression, which led to the transcriptional promotion of HDAC4 driving Notch1 signaling in podocyte inflammation in response to high fructose. Our findings suggested that magnolol may be a potential compound to ameliorate high fructose-induced podocyte inflammatory damage, at least in part, through inhibiting TKFC/Sp1/HDAC4/Notch1 activation. More meaningfully, magnolol improved the lesion of glomerular filtration function induced by high fructose, providing new evidence for its potential role in podocyte protection.

## Figures and Tables

**Table 1:** Primer sequences utilized for qRT-PCR (homo sapiens).

Gene	Sequences (5′~3′)
*TKFC*	Forward: ACTGGGATCGGCTCAACT Reverse: CACCTTGTGTATAAGCACCGT
*TNF-α*	Forward: CCTGCTGCACTTTGGAGTGA Reverse: GAGGGTTTGCTACAACATGGG
*HDAC4*-promoter	Forward: TCCAGCAGCCAATGAGGTCC Reverse: TTCTCCCCACTCCAGCGTCG
*β-actin*	Forward: CTACCTCATGAAGATCCTCACCGA Reverse: TTCTCCTTAATGTCACGCACGATT

**Table 2:** siRNA sequences (homo sapiens) used in this study.

siRNA	Sequences (5′~3′)
*TKFC* siRNA	Forward: CCUUCACUGUCCUGAAGAAdTdT Reverse: UUCUUCAGGACAGUGAAGGdTdT
Control siRNA	Forward: UUCUCCGAACGUGUCACGUdTdT Reverse: ACGUGACACGUUCGGAGAAdTT

## Data Availability

Data are included within the article.
